# Coexistence of Histologically Confirmed Hashimoto's Thyroiditis with Different Stages of Papillary Thyroid Carcinoma in a Consecutive Chinese Cohort

**DOI:** 10.1155/2014/769294

**Published:** 2014-11-18

**Authors:** Xiaoyun Liu, Lijun Zhu, Dai Cui, Zhixiao Wang, Huanhuan Chen, Yu Duan, Meiping Shen, Yunsong Wu, Rong Rong, Zhihong Zhang, Xiaodong Wang, Jiawei Chen, Erik K. Alexander, Tao Yang

**Affiliations:** ^1^Department of Endocrinology, First Affiliated Hospital of Nanjing Medical University, Nanjing, Jiangsu 210029, China; ^2^Department of Children's Health Care, Nanjing Maternity and Child Health Care Hospital Affiliated to Nanjing Medical University, Nanjing, Jiangsu 210029, China; ^3^Department of Surgery, First Affiliated Hospital of Nanjing Medical University, Nanjing, Jiangsu 210029, China; ^4^Department of Pathology, First Affiliated Hospital of Nanjing Medical University, Nanjing, Jiangsu 210029, China; ^5^Thyroid Unit, Division of Endocrinology, Metabolism and Diabetes, Department of Medicine, Brigham & Women's Hospital and Harvard Medical School, Boston, MA 02115, USA

## Abstract

*Purpose*. To determine the relationship between Hashimoto's thyroiditis (HT) and all stages of papillary thyroid carcinoma (PTC) with or without local lymph node metastasis (LNM). *Methods*. We conducted a retrospective study of thyroidectomies from 2008–2013 in First Affiliated Hospital of Nanjing Medical University. We categorized patients according to the presence of histopathologically proven HT. The prevalence of mPTC (maximum diameter ≤ 10 mm) and crPTC (clinical relevant PTC) and local LNM rates were compared. *Results*. We evaluated 6,432 consecutive thyroidectomies. In total, 1,328 specimens were confirmed as HT. The prevalence of PTC in this HT cohort was 43.8%, significantly higher than non-HT group. After adjustment of gender and age, the prevalence of PTC was still higher in HT group. HT was a risk factor for PTC in multivariate analysis with odds ratio 2.725 (95% CI, 2.390–3.109) (*P* < 0.001). However, no correlation was found between HT and LNM of PTC. *Conclusion*. HT was associated with an increased prevalence of all stages of PTC, independent of tumor size, gender, and age. In contrast, locally advanced disease defined by LNM was unrelated to HT. These data suggest an association of HT with low risk PTC and a potential protective immunologic effect from further disease progression.

## 1. Introduction

Thyroid cancer is the most common endocrine malignancy worldwide, with a significant increase in global incidence [1–4] over the past decade. This is true both in the United States [2, 5] and in eastern Asia including China [6–9]. About 70% to 80% of thyroid cancers are papillary thyroid carcinoma (PTC) [9, 10]. Despite surgical advances and better long-term results, however, the underlying pathogenesis of PTC remains unclear.

Hashimoto's thyroiditis (HT) has been postulated to have a causative relationship to PTC, being suggested as a possible risk factor for developing PTC. This link was first proposed in 1955 with several subsequent retrospective studies showing similar results [11–15]. The exact mechanism remains uncertain. Some studies suggest that stimulating TSH concentrations (often mildly elevated) may be one responsible factor for this relationship [16]. In support, treatment with L-T_4_ may reduce TSH levels and decreases the prevalence of clinically detectable PTC [12]. However, other studies are contradictory finding TSH concentration similar among patients regardless of PTC or benign disease [17].

It is well known that PTC represents a spectrum of disease inclusive of heterogeneity and severity in histologic patterns and/or tumor stages. Occult or microscopic PTC (mPTC) is frequently incidentally detected in histologic samples even when benign nodules are assumed. It is uncertain whether incidental micro-PTC (mPTC) simply represents an early stage of overt (or clinically relevant) PTC or is actually a different subtype of PTC which seldom progresses. It is therefore reasonable to postulate that the analysis of PTC as a unified single group may in part be responsible for the conflicting published data. Notably, most previous studies only evaluated clinically relevant PTC (crPTC, maximum diameter > 1 cm in size). We hypothesize that mPTC might be the early stage of crPTC with similar relationships existing between mPTC and HT when compared to crPTC and HT. However, some evidence suggests that the presence of HT may be associated with less risk of PTC lymph node metastasis (LNM) and an overall better prognosis [11, 18]. To elucidate the uncertainty as to the influence of HT upon local tumor growth (even from a very early stage), we conducted a retrospective study trying to clarify the relationship between HT and different stages of PTC.

## 2. Methods

We collected data from the Register and Reporting system in Department of Pathology, the First Affiliated Hospital of Nanjing Medical University. Particularly, we reviewed all consecutive thyroid histopathological reports from January 2008 to December 2013. Patient demographic data was obtained by chart review at a central location by Xiaoyun Liu, Lijun Zhu, Dai Cui, Zhixiao Wang, Huanhuan Chen, Yu Duan, Xiaodong Wang, and Jiawei Chen.

Generally, thyroidectomies were performed for the following reasons: (a) worrisome findings from ultrasonography and/or abnormal lymph node enlargement, (b) malignancy suspected from previous thyroid FNA, or inconclusive FNA results, (c) patients with multiple or bilateral nodules or symptoms of neck or throat compression, or enlargement during follow-up, and (d) concerning clinical or physical examination findings warranting consideration for removal. A small minority of patients chose thyroidectomy at their own wish, primarily for cosmetic reasons.

Malignancy, if present, was reported as one of the following subtypes: crPTC, with the maximum diameter larger than 10 mm; mPTC, with the maximum diameter less than or equal to 10 mm [19, 20]; follicular thyroid carcinoma (FTC); medullary thyroid carcinoma (MTC); and anaplastic thyroid carcinoma (ATC). For each case, we collected data confirming the maximum diameter of the tumor, tumor multifocality, and other pathologic features. The impact of Hashimoto's thyroiditis upon mPTC and crPTC rates was the primary focus of this investigation. Papillary structures sometimes seen in nodular hyperplasia were not recorded as PTC. All pathological findings were confirmed by three experienced endocrine pathologists (Yunsong Wu, Rong Rong, and Zhihong Zhang).

Lymph node dissection was performed at the discretion of the surgeon based upon clinical and other factors according to the guideline from the American Thyroid Association (ATA) [21]. Most often, a neck ultrasound or computed tomography (CT) scan was performed prior to surgery allowing assessment of neck adenopathy. Frozen sections were routinely used to guide the extent of the surgical procedures. If the nodule was found to be malignant by frozen section and no abnormal lymph nodes were identified on preoperative imaging (or during the surgery), an ipsilateral central lymph node dissection (CLND) from level VI was generally performed. Only when the tumor size was within 10 mm and no abnormal lymph node was found during the operation, CLND was not routinely performed. If abnormal lymph nodes were identified during preoperative ultrasound or intraoperatively, and the snap-frozen sample showed malignant lesion, a modified lateral lymph node dissection (LLND) was performed. All the samples were formalin fixed paraffin embedded (FFPE) for final histopathology confirmation.

The histologic diagnosis of HT was made according to accepted standards [18]. HT was defined as the presence of diffuse lymphocytic infiltration with the formation of lymphoid follicles and the presence of reactive germinal centers; the infiltrate had to occur in a normal region of the thyroid gland, distinct from the site of the thyroid carcinoma. Peritumoural inflammatory response and small areas of lymphocytic infiltration were not designated as HT. The diagnosis of HT was solely made upon the histological findings. The antithyroglobulin and antithyroid peroxidase antibody levels were not routinely measured for the diagnosis of HT.

We recruited 6,432 cases with valid pathology reports including all thyroidectomies performed over the six-year period. We next categorized each according to the histologic diagnosis of HT and separately for the identification of mPTC and crPTC (or other cancer types).

Quantitative data are shown as mean ± SD, compared using independent samples *t*-test, whereas numbers and percentage are provided for qualitative data. Percentages were compared using the *χ* ^2^ test. The odds ratio (OR) and the 95% confidence interval (CI) for relationships between each variable and PTC or positive LNM (yes or no) were calculated using binary logistic regression. All tests were 2-sided, and a *P* value <0.05 was considered statistically significant. Statistical analyses were performed with SPSS software, version 13.0 for Windows (SPSS Inc., Chicago, IL, USA).

This study was reviewed and deemed exempt from written informed consent by the Institutional Review Board (IRB) of the First Affiliated Hospital of Nanjing Medical University. It was approved by the IRB for analysis.

## 3. Results

### 3.1. Basic Characteristics and Thyroid Carcinoma Distribution of Non-HT and HT Groups

Over a 6-year period, we evaluated 6,432 consecutive patients who underwent thyroidectomy. Demographic and pathologic details for all subjects are shown in Table 1. Among this cohort, 20.6% (1,328/6,432) of our thyroidectomy population was histologically confirmed to have HT. Not surprisingly, those with HT were disproportionately female, consistent with other autoimmune illnesses. The mean age of the HT cohort was 46.2 ± 13.6 years, significantly younger than the non-HT group.

A total of 1,977 thyroidectomy specimens (30.7% of 6,432) were shown to harbor malignancy—mostly PTC (87.1% of all malignancy). However, 823 cases (41.6% of all malignancy) were best classified as mPTC and 899 specimens (45.5% of all malignancy) demonstrated a clinically relevant (>1 cm) papillary thyroid carcinoma. A low but detectable rate of FTC, MTC, and ATC was also identified, but these rates were not different in the HT cohort versus others (Table 1).

We next determined the prevalence of thyroid malignancy in thyroidectomy specimens with or without histologic evidence of HT. In the HT group, 46.9% specimens were found to also contain a thyroid malignancy of any pathological type, compared to 26.5% of those without HT (*P* < 0.01, Table 1). Interestingly, however, the difference in malignancy rate was solely attributable to differences in the proportion of PTC in the two cohorts. Of the 823 patients with mPTC, 541 (10.6%) patients were in the non-HT group while 282 (21.2%) patients were in HT group (Table 1). This finding was consistent for crPTC as well. No difference was detected in the rate of FTC, MTC, or other malignancies (Table 1).

### 3.2. Prevalence of PTC (including mPTC and crPTC) in Various Subgroups

**Figure 1:**
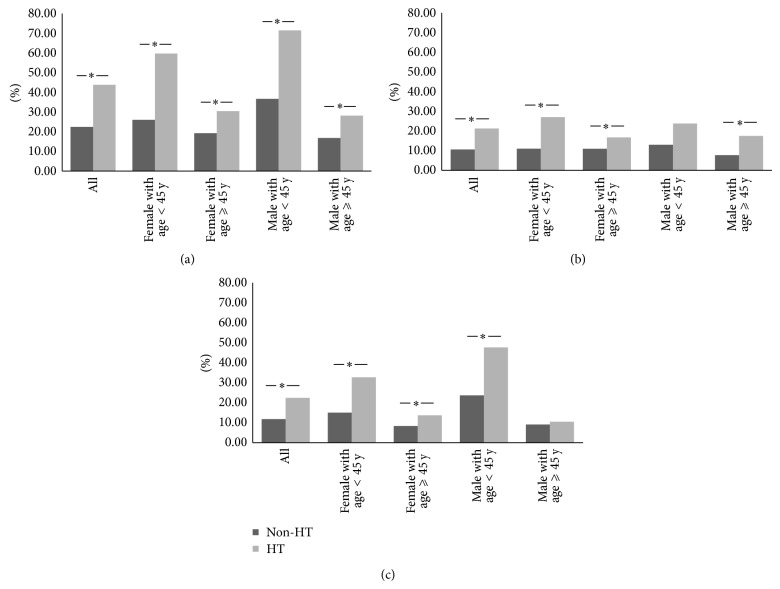
The prevalence of PTC (a), mPTC (b), and crPTC (c) in different age and gender groups. ^*^ *P* < 0.05.

Due to the gender and age discrepancy in non-HT and HT group, we divided the total population into four subcategories according to the sex and age differences. It was shown consistently that the prevalence of PTC was higher in all Hashimoto's cohorts in comparison with non-HT groups (Figure 1(a) and supplementary Table 1  in Supplementary Material available online at  http://dx.doi.org/10.1155/2014/769294). Nearly all subgroups analysis showed similar statistical findings for the prevalence of mPTC with the exception of male under the age of 45 years (Figure 1(b) and supplementary Table 1, *P* = 0.05) and for the prevalence of crPTC with one exception in male ⩾45 years (Figure 1(c) and supplementary Table 1).

### 3.3. Risk Factor for PTCs

We used univariate and multivariate analysis to determine whether the presence of HT would be an independent risk factor for PTC. As it was shown in Table 2, HT was a significant independent risk factor for PTC with odds ratio 2.725 (95% CI, 2.390–3.109). The age ⩾45 years was a protective risk factor for PTC with odds ratio 0.474 (95% CI, 0.423–0.532).

### 3.4. Local Lymph Node Metastasis (LNM) between Groups

**Figure 2:**
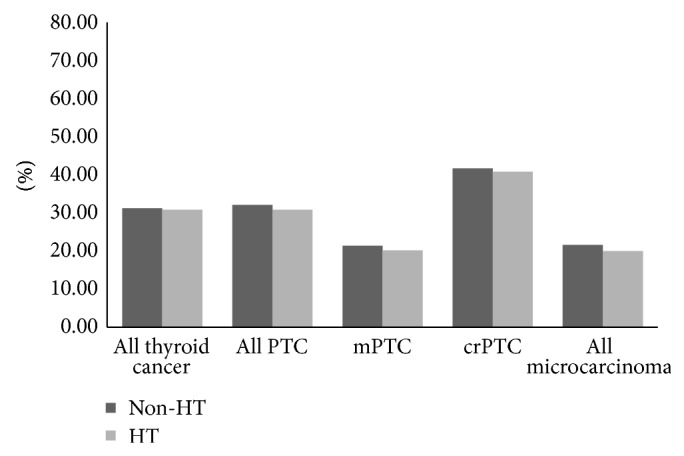
Local lymph node metastasis in different groups. No significance was found between HT and non-HT groups.

We next investigated the association of histologic HT upon local metastatic disease as defined by lymph node metastasis (LNM). Inclusive of all thyroid malignancy identified over 6 years, the presence of HT showed a neutral effect upon local LNM when thyroid cancer was present (*P* > 0.05, Figure 2 and supplementary Table 2).

### 3.5. Risk Factors for LNM in PTCs

We then determined the risk factors of LNM in PTCs. As shown in Table 3, we found that male gender, age < 45 years, foci of tumor, and maximum diameter were risk factors for LNM in PTCs, while HT was neither a protective nor risk factor for LNM in PTCs with OR 0.978, 95% CI (0.772–1.239) (Table 3).

### 3.6. Basic Characteristics of All PTCs between Non-HT and HT Groups

All cohorts evaluated with PTC demonstrated an association with younger age and female predominance in the Hashimoto's cohort compared with non-HT groups. This was independent of tumor size (Table 4). No difference was noted in the number of positive lymph nodes or the number of specific tumor foci between HT and non-HT groups, respectively. Interestingly, in all patients with Hashimoto's disease, the size of all identified clinically relevant (crPTC) was generally smaller compared to those without Hashimoto's disease. In contrast, mPTCs were more often bilateral (though not more often multifocal) in the HT cohort compared to non-HT cohort.

## 4. Discussion

The relationship between Hashimoto's thyroiditis and thyroid malignancy has long been controversial. Differences in study design, selection bias, and standardized approaches have led to a mixed literature in this regard. Arguably, to best investigate this important association, a large cohort of consecutive patients must be studied and all diagnoses—such as Hashimoto's disease and thyroid carcinoma—defined by expert histopathologic analysis.

In this regard, we evaluated 6,432 consecutive thyroidectomies over 6 years from a single referral institution in China, revealing a unique association between Hashimoto's thyroiditis and PTC. Particularly, Hashimoto's thyroiditis increases the risk of papillary thyroid carcinoma in affected subjects, though it appears to impart no risk factor upon lymph node metastasis and progressive disease. This association was true both for mPTC and for crPTC, though it was not detected for all other thyroid malignancies including follicular, medullary, and anaplastic thyroid carcinoma. These data support previous pilot findings and suggest those with HT may warrant increased attention for the development of clinically relevant thyroid nodules given their higher risk of malignancy.

A recent meta-analysis of this topic similarly demonstrated that Hashimoto's thyroiditis is correlated to the presence of PTC, with carcinoma detected in 9.46~36.60% of patients with HT. In our study, the prevalence of PTC in the HT cohort was 43.8%, while in the non-HT group the prevalence was 22.4%. Zhang showed in areas where the prevalence of HT was even higher the prevalence of PTC in HT was also higher, reaching 58.3% compared to 44.3% in non-HT group in the same region [22]. This suggests a similar influence of Hashimoto's disease upon cancer risk across different populations despite variable incidence of malignancy. Our study showed that HT was not only closely related to an increased risk of crPTC, but also significantly related to the risk of mPTC. This similar relationship to both crPTC and mPTC strengthens the translatability of our data and lessens concern for selection bias in our study population. Beyond its clinical importance, our data also lends support to the hypothesis that microcellular dynamics (such as inflammation) impact the development of thyroid malignancy independent of nodule size.

Interestingly, although the coexistence of Hashimoto's disease appears related to increased risk of PTC, our results showed HT was not a risk factor for LNM in PTCs. And the maximum diameter of PTC seemed to be related to HT in mPTC stage and conversely in the crPTC stage. The mechanism behind this observation remains uncertain. In support, however, Dvorkin has previously found that differentiated thyroid cancer is similarly associated with less aggressive disease and better outcome in patients with coexisting HT, while Zhou has also found that HT is not a risk factor for occult contralateral carcinoma in patients with unilateral mPTCs [23]. Increasingly, clinical and basic investigations appear to confirm the important influence of the body's immune system in relation to malignant disease. While infection and immune response have been associated with an increased risk of malignancy in illnesses such as hepatitis, it is equally clear that immunologic influence can also positively influence malignant progression. This is perhaps best exemplified by melanoma or breast carcinoma, where numerous case reports have described unusual, prolonged remissions or late recurrences which implicate the immune system as a causative factor. Thus, while speculative, it is not unreasonable to postulate that chronic inflammation of the thyroid may increase malignant transformation, while nonetheless stimulating an immunologic milieu in which tumor expansion and malignant growth are less favored. Clinically, such a hypothesis would be nearly impossible to prove, as there currently exist no immunomodulating therapies for Hashimoto's disease by which one could study cancer prevalence and progression following a randomized intervention. However, it is also possible that HT may be the “response” to the tumor in genetically predisposed subjects and that this response may slow down cancer progression. Regardless, these data nonetheless provide insight into the complex interactions of thyroid cellular biochemistry and immunology and provide a stimulus for further investigation. We did find that male gender, age < 45 years, foci of tumor, and maximum diameter were risk factors for LNM in PTCs in accordance with another previous study [24].

The strength of our study relates to the size and uniformity of our study cohort, as well as the strict histopathologic definitions used for both Hashimoto's thyroiditis and thyroid malignancy. We also analyzed PTC across the full spectrum of size, including evaluation of microcarcinomas no more than 1 cm in diameter. The similar relationship of HT to both mPTC and crPTC is notable and speaks against selection or sampling bias. Nonetheless, we acknowledge the limitations to our investigation. Our study was retrospective and from a single study center. We tried to eliminate further selection bias by subanalyzing our total cohort by gender, age, size of tumor, and using binary logistic regression method. We also note that a relatively large percent (69.3%) of benign nodules was resected. Most often, this was simply due to nodule size more than 3 cm or the presence of large multinodular goiters (data not shown). While an ideal study would prospectively investigate thyroidectomy histopathology among all patients with nodular disease, it is unreasonable to routinely perform thyroidectomies on all low risk patients [21], or those with benign disease. We also note that HT itself was not a thyroidectomy indication in our study. Recent data confirm modest interobserver variability in cancer diagnosis [25], which could reasonably suggest slight differences in translation of these data at other institutions.

In conclusion, these data provide the most extensive consecutive analysis of the relationship between Hashimoto's thyroiditis and thyroid carcinoma, confirming an increased risk of PTC in the setting of HT. While this increased risk appears independent of PTC size, however, during more advanced stage, HT was no longer a risk factor suggesting a potential protective effect of the inflammatory milieu upon the progression of malignancy as defined by local LNM. These data support previous pilot findings and suggest that patients with HT may warrant increased attention for the development of clinically relevant thyroid nodules given their higher risk of malignancy.

## Supplementary Material

Description for Supplementary Table 1: Description: It was shown consistently that the prevalence of PTC was higher in all HT cohorts in comparison with non-HT groups (P<0.05). Nearly all subgroups analysis showed similar statistical findings for the prevalence of mPTC with the exception of male < 45 years (P = 0.05) and for the prevalence of crPTC as well with one exception in male ≥ 45 years (P=0.72).
Description for Supplementary Table 2: Description: Inclusive of all thyroid malignancy identified over the six years, the presence of HT showed a neutral effect upon local LNM when thyroid cancer was present (P > 0.05).

## Figures and Tables

**Table 1:** Baseline characteristics and thyroid carcinoma distribution between non-HT and HT groups.

	Non-HT (*n* = 5104)	HT (*n* = 1328)	*χ* ^2^	*P*
Age at diagnosis	48.72 ± 13.74	46.21 ± 13.55		<0.01
<45 y (*n* = 2565)	1972 (38.6%)	593 (44.7%)	15.914	<0.01
*⩾*45 y (*n* = 3867)	3132 (61.4%)	735 (55.3%)		
Gender				
Male (*n* = 1373)	1274 (25.0%)	99 (7.5%)	192.350	<0.01
Female (*n* = 5059)	3830 (75.0%)	1229 (92.5%)		
PTC (*n* = 1722)	1141 (22.4%)	581 (43.8%)	246.051	<0.01
mPTC (*n* = 823)	541 (10.6%)	282 (21.2%)	106.826	<0.01
crPTC (*n* = 899)	600 (11.8%)	299 (22.5%)	101.467	<0.01
FTC (*n* = 115)	97 (1.9%)	18 (1.4%)	1.783	0.18
MTC (*n* = 34)	26 (0.5%)	8 (0.6%)	0.173	0.68
ATC (*n* = 4)	3 (0.1%)	1 (0.1%)	0.046	0.83
MC (*n* = 841)	556 (43.9%)	285 (46.6%)	1.204	0.27
Other cancer (*n* = 101)	86 (1.7%)	15 (1.1%)	2.103	0.15
All cancer (*n* = 1977)	1354 (26.5%)	623 (46.9%)	205.683	<0.01
Total (*n* = 6,432)	**5104**	**1328**		

Data are expressed as number (percentage) or mean ± SD unless otherwise specified.

**Table 2:** Univariate and multivariate analysis for PTC.

Independent variable	Univariate		Multivariate	
	OR (95% CI)	*P* value	OR	*P* value
Age (*⩾*45 versus <45)	0.469 (0.420–0.525)	0.001	0.474 (0.423–0.532)	0.001
Gender (male versus female)	0.906 (0.790–1.039)	0.157	1.153 (0.999–1.330)	0.051
Hashimoto thyroiditis (HT versus non-HT)	2.701 (2.380–3.067)	0.001	2.725 (2.390–3.109)	0.001

**Table 3:** Univariate and multivariate analysis for LNM in PTCs.

Independent variable	Univariate		Multivariate	
	OR (95% CI)	*P* value	OR (95% CI)	*P* value
Age (*⩾*45 versus <45)	0.439 (0.355–0.542)	0.001	0.455 (0.363–0.569)	0.001
Gender (Male versus Female)	1.723 (1.351–2.197)	0.001	1.544 (1.181–2.017)	0.001
Hashimoto thyroiditis (HT versus non-HT)	0.943 (0.760–1.170)	0.593	0.978 (0.772–1.239)	0.852
Foci of tumor	1.547 (1.367–1.750)	0.001	1.471 (1.293–1.673)	0.001
Maximum diameter	1.047 (1.038–1.057)	0.001	1.041 (1.031–1.051)	0.001

**Table 4:** Baseline data of PTC (mPTC and crPTC) between non-HT and HT group.

	Non-HT	HT	Total	*P*
All size of PTC (*n*)	1141	581	1722	
Age at diagnosis	45.25 ± 13.63	41.40 ± 13.26		<0.01
<45 y	560 (49.1%)	359 (61.8%)	919 (53.4%)	<0.01
*⩾*45 y	581 (50.9%)	222 (38.2%)	803 (46.6%)	
Gender				
Male	301 (26.4%)	46 (7.9%)	347 (20.2%)	<0.01
Female	840 (73.6%)	535 (92.1%)	1375 (79.8%)	
Number of positive lymph nodes	3.05 ± 0.192	2.66 ± 0.235		0.21
Foci of tumor	1.46 ± 0.025	1.46 ± 0.032		0.91
Maximum diameter (mm)	15.08 ± 0.358	13.92 ± 0.421		0.05
Bilateral tumor	241 (21.2%)	140 (24.1%)	381	0.16
Positive LNM	366 (32.1%)	179 (30.8%)	545	0.60

mPTC (*n*)	541	282	822	
Age at diagnosis	47.21 ± 12.03	43.09 ± 13.04		<0.01
<45 y	226 (41.8%)	159 (56.4%)	385	<0.01
*⩾*45 y	315 (58.2%)	123 (43.6)	438	
Gender				
Male	121 (22.4%)	20 (7.1%)	141	<0.01
Female	420 (77.6%)	262 (92.9%)	682	
Number of positive lymph nodes	1.71 ± 0.196	1.46 ± 0.237		0.42
Foci of tumor	1.32 ± 0.030	1.40 ± 0.043		0.11
Maximum diameter (mm)	6.02 ± 0.122	6.43 ± 0.165		0.05
Bilateral tumor	66 (12.2%)	52 (18.4%)	118	0.02
Positive LNM	116 (21.4%)	57 (20.2%)	173	0.68

crPTC (*n*)	600	299	899	
Age at diagnosis	43.48 ± 14.72	39.80 ± 13.29		<0.01
<45 y	334 (55.7%)	200 (66.9%)	534	<0.01
*⩾*45 y	266 (44.3%)	99 (33.1%)	365	
Gender				
Male	180 (30.0%)	26 (8.7%)	206	
Female	420 (70.0%)	273 (91.3%)	693	<0.01
Number of positive lymph nodes	3.97 ± 0.285	3.62 ± 0.363		0.46
Foci of tumor	1.59 ± 0.039	1.51 ± 0.046		0.20
Maximum diameter (mm)	23.26 ± 0.467	20.98 ± 0.549		<0.01
Bilateral tumor	175 (29.3%)	88 (29.5%)	263	0.93
Positive LNM	250 (41.7%)	122 (40.8%)	372	0.80

Data are expressed as number (percentage) or mean ± SD.
