# Supplementary material for: Coexistence of Histologically Confirmed Hashimoto's Thyroiditis with Different Stages of Papillary Thyroid Carcinoma in a Consecutive Chinese Cohort
Source: Int J Endocrinol. 2014 Nov 18;2014:769294. doi: 10.1155/2014/769294 (PMC4255062; doi:10.1155/2014/769294)
Supplement: Supplementary file 1 — Description for Supplementary Table 1: Description: It was shown consistently that the prevalence of PTC was higher in all HT cohorts in comparison with non-HT groups (P<0.05). Nearly all subgroups analysis showed similar statistical findings for the prevalence of mPTC with the exception of male < 45 years (P = 0.05) and for the prevalence of crPTC as well with one exception in male ≥ 45 years (P=0.72). Description for Supplementary Table 2: Description: Inclusive of all thyroid malignancy identified over the six years, the presence of HT showed a neutral effect upon local LNM when thyroid cancer was present (P > 0.05). [file 769294.f1.pdf]

Supplementary Table 1. Prevalence of PTC (mPTC and crPTC) between Non-HT and HT groups matched by gender and age

	**Non-HT (n=**5104)	**HT (n=**1328)	χ^2	***P* Value**
All PTC(n=1722)	1141(22.4%)	581(43.8%)	246.051	<0.01
Female with Age<45y(n=2084)	399(26.0%)	329(59.7%)	202.303	<0.01
Female with age>=45y(n=2975)	441(19.2%)	206(30.4%)	38.479	<0.01
Male with age<45y(n=481)	161(36.7%)	30(71.4%)	19.339	<0.01
Male with age>=45y(n=892)	140(16.8%)	16(28.1%)	4.725	0.03
mPTC(n=823)	541(10.6%)	282(21.2%)	106.826	<0.01
Female with Age<45y(n=2084)	169(11.0%)	149(27.0%)	80.421	<0.01
Female with age>=45y(n=2975)	251(10.9%)	113(16.7%)	16.058	<0.01
Male with age<45y(n=481)	57(13.0%)	10(23.8%)	3.747	0.05
Male with age>=45y(n=892)	64(7.7%)	10(17.5%)	6.845	<0.01
crPTC(n=899)	600 (11.8%)	299(22.4%)	101.467	<0.01
Female with Age<45y(n=2084)	230(15.0%)	180(32.7%)	80.031	<0.01
Female with age>=45y(n=2975)	190(8.3%)	93(13.7%)	18.032	<0.01
Male with age<45y(n=481)	104(23.7%)	20(47.6%)	11.471	<0.01
Male with age>=45y(n=892)	76(9.1%)	6(10.5%)	0.130	0.72

Data are expressed as number (percentage).

Supplementary Table 2. LNM comparison of different cancer types between Non-HT and HT groups

LNM	Non-HT	HT	Total	χ^2	***P* Value**
All thyroid cancer	423(31.2%)	192(30.8%)	615(31.1%)	0.035	0.85
All-PTC	366(32.1%)	179(30.8%)	545(31.6%)	0.286	0.59
mPTC	116(21.4%)	57(20.2%)	173(21.0%)	0.169	0.68
crPTC	250(41.7%)	122(40.8%)	372(41.4%)	0.061	0.80
All microcarcinoma	120(21.6%)	57(20.0%)	177(21.0%)	0.284	0.59
FTC	4(4.1%)	0(0.0%)	4(3.5%)	0.769	0.38
Medullary Carcinoma	10(38.5%)	5(62.5%)	15(44.1%)	1.434	0.23
Anaplastic Carcinoma	1(33.3%）	1(100.0%)	2(50%)	1.333	0.25
Other cancer	42(48.8%)	7(46.7%)	49(48.5%)	0.024	0.88

Data are expressed as number (percentage).
